# Distal radius anatomy applied to the treatment of wrist fractures by plate: a review of recent literature

**DOI:** 10.1051/sicotj/2015012

**Published:** 2015-06-19

**Authors:** Laurent Obert, François Loisel, Nicolas Gasse, Daniel Lepage

**Affiliations:** 1 Orthopaedic, Traumatology, Plastic & Hand Surgery Unit, University Hospital of Besançon 25000 Besançon France; 2 Intervention, Innovation, Imagery, Engineering in Health (EA 4268), Medical and Pharmacology Section, IFR 133, University of Franche-Comté 25000 Besançon France; 3 Clinique Saint Vincent 40 chemin des Tilleroyes 25044 Besançon Cedex France

**Keywords:** Distal radius, Anatomy, Volar plate

## Abstract

Few studies on the anatomy of the radial epiphysis have been published in the past 10 years. However, with the availability of new intra- and extra-medullary implants and the recent rash of avoidable iatrogenic injuries, now is the time for a more detailed description of the metaphyseal-epiphyseal regions in the distal radius. Published studies on distal radius anatomy in recent years have focused on three aspects: distal limit and watershed line, dorsal tubercle, and wrist columns. Furthermore, a fresh look at distal radius biomechanics shows that the loads experienced by the distal radius vary greatly. This information should be taken into account during volar plating of distal radius fractures.

## General features of distal radius anatomy

The distal portion of the radius has a quadrilateral cross-section and includes the metaphyseal and epiphyseal regions. Anatomic features of the distal radius include the styloid process, the dorsal tubercle, and four surfaces: anterior, lateral, posterior, and medial. The scaphoid fossa, lunate fossa, and sigmoid notch are three concave articular surfaces. The scaphoid fossa and the lunate fossa are separated by a dorsal-volar ridge which defines the scaphoid and lunate facets.

**Figure 1.**
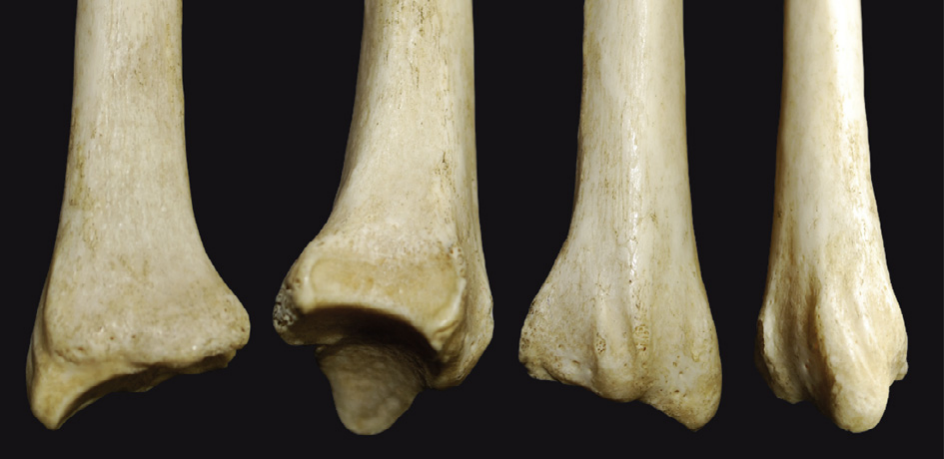
The four surfaces (anterior, medial, posterior, and lateral) of the distal radius are shown from left to right, along with the styloid process and dorsal tubercle.

The anterior surface is concave, angled anteriorly, and covered by the pronator quadratus (Figure 1). Its rough surface provides an attachment point for the palmar radiocarpal ligaments. The anterior surface extends radially from the radial styloid ulnarly to the triangular fibrocartilage complex (TFCC). It extends distally and ulnarly to the capitate (radiocapitate), lunate (radiolunate), and triquetrum (radiotriquetral).

The lateral surface extends along the lateral margin to form the styloid process (Figure 1). The styloid process is conical and projects 10–12 mm beyond the articular surface for the proximal scaphoid and lunate. The distal part of the styloid provides an attachment for the articular capsule and for the capsular thickening of the collateral ligament. A more proximal area at the base of the styloid provides the brachioradialis attachment. The radial styloid area may have a flat groove for the tendon of the first dorsal compartment (abductor pollicis longus and extensor pollicis brevis tendons).

The posterior surface of the distal radius is irregular, convex and acts as a fulcrum for the extensor tendon (Figure 1). A prominent dorsal tubercle (Lister’s tubercle) lies 5–10 mm from the distal joint surface. There is a smooth groove for passage of the extensor pollicis longus (EPL) tendon on the medial aspect of the dorsal tubercle.

The medial surface of the distal radius consists of the ulnar notch and the articular surface for the ulnar head (Figure 1). The distal radius rotates about the ulnar head by means of the sigmoid notch, which is concave with well-defined dorsal, palmar, and distal margins. Its depth varies according to the articulation with the ulnar head. Ulnar length varies with radial length and changes with pronation and supination. There are various degrees of positive or negative ulnar variance which affect the amount of force transmitted to the distal radius and to the TFCC. Between the distal radioulnar joint and the radiocarpal joint there is a ridge, located in the ulnar notch; this ridge provides the radial attachment point for the TFCC. At various radioulnar deviations there may be greater or lesser contact with the TFCC. The distal articular surface of the radius has a radial inclination averaging 22° (21–25°) and an average volar tilt of 11° (2–20°). The sigmoid notch angles distally and medially an average of 22°.

There are three important dorsal ligaments in the wrist; two of the three – the radiolunate and the radiotriquetral – extend distally and ulnarly from the distal radius to attach to the proximal carpal row. Ulnarly to the dorsal tubercle, there are grooves for passage of the extensor indicis, which passes deeper than the extensor digitorum communis. The posterior interosseous nerve runs along the dorsal margin, next to the cortex.

## A fresh look at distal radius anatomy

Few studies on the anatomy of the radial epiphysis have been published in the past 10 years [1]. However, with the availability of new intra- and extra-medullary implants and the recent rash of avoidable iatrogenic injuries, now is the time for a more detailed description of the metaphyseal-epiphyseal regions in the distal radius. Studies of this iconic, yet still misunderstood area are few and far between. One example is the study by Herzberg et al. [2], who showed that the volar cortex was thicker than the dorsal one. Published studies on distal radius anatomy in recent years have focused on three aspects: distal limit and watershed line, dorsal tubercle, and wrist columns.

### Watershed line concept (Figure 2)

Windisch et al. [3] were the first to describe an area where the capsule inserts between two lines and two contours on the distal radius. A few years later he published another paper describing a protuberance which he called the promontory of the radius. The geometry of this protuberance varies greatly [4]. In 2005, Nelson introduced the concept of the watershed line by describing the most distal line on the radius. He also described a more proximal line which corresponds to the distal part of the pronator quadratus (Figure 3) [5]. The pronator quadratus line marks the highest part of the epiphysis and helps the surgeon visualize the patient-specific radius curvature. If an implant goes beyond this line when viewed on lateral X-rays, there is potential for impingement with the thumb and finger flexor tendons. The watershed line marks the most distal edge of the epiphysis; sometimes it is as high as the pronator quadratus line, sometimes it is higher. A small 3–5 mm thick strip of bone separates these two lines. Going past the watershed line will land you in the joint!

**Figure 2.**
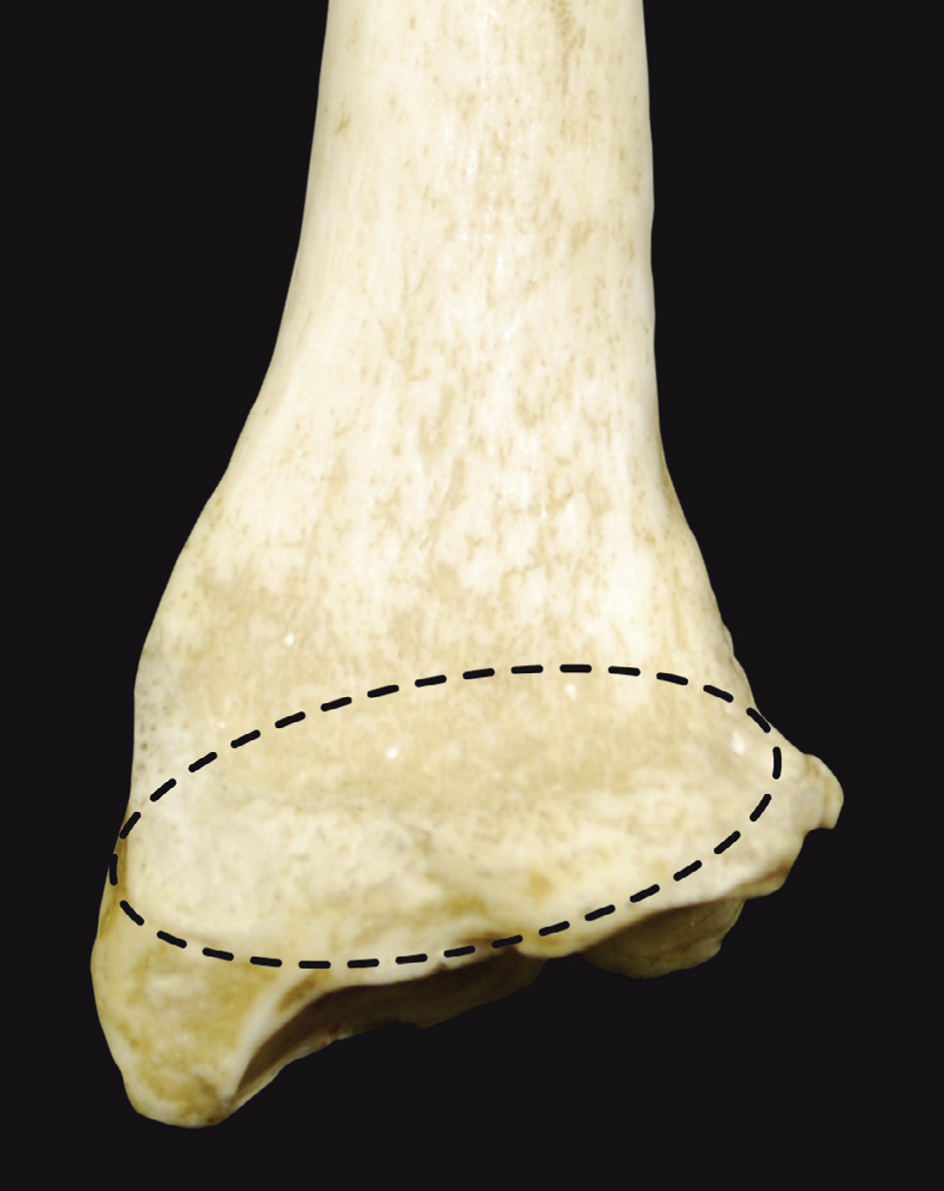
Volar view of the distal radius showing, in dotted line, the anatomical region of interest.

**Figure 3.**
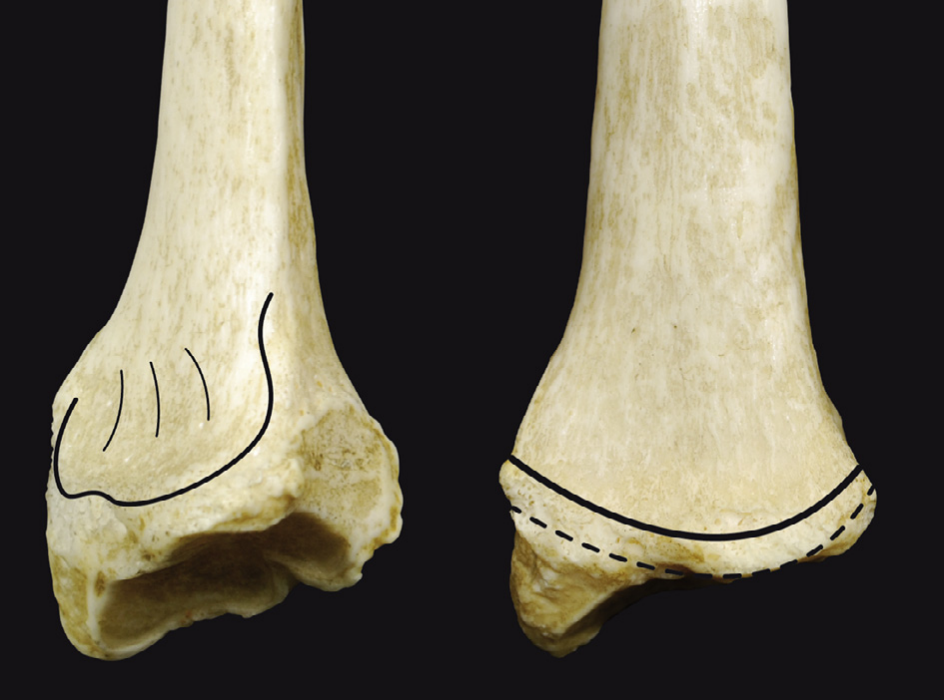
One oblique and one anterior view of the distal radius showing the more proximal pronator quadratus line (solid line) and the watershed line (dashed line).

**Figure 4.**
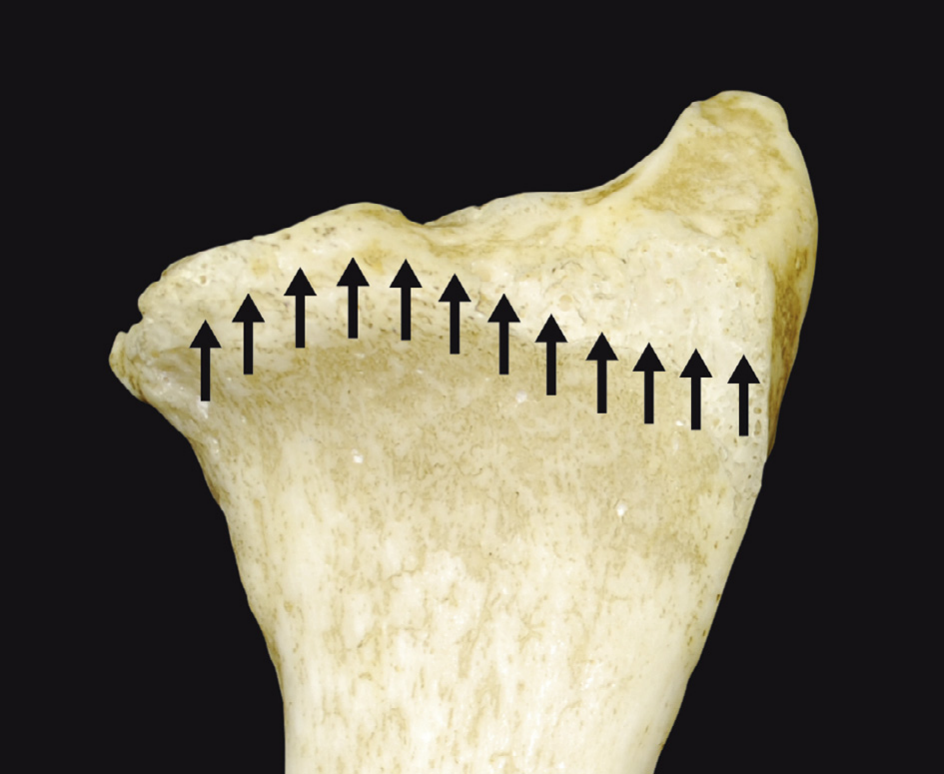
The area where a plate is applied is more ulnar than radial. Plate design should be based on this principle, and should not be too radial because the ulnar column is further forward than the radial column.

Gasse et al. [6] confirmed the presence of these two easily recognizable lines on 70 distal radius specimens from cadavers. The ulnar column was found to be longer distally than the radial column and the radial styloid process was not in the distal radius plane (Figure 4). Imatani et al. [7] studied the volar aspect of the distal radius in 20 distal forearms from 10 cadavers macroscopically and histologically. The watershed line and pronator quadratus line were present in only four of these specimens. A medial bony prominence was also found on the watershed line. These findings suggest that the watershed line may not be a distinct line; it may correspond instead to the distal margin of the pronator fossa in the lateral half of the volar radius and to a hypothetical line between the distal and proximal lines in the medial half [7]. The differences between the Imatani and Grasse studies may be due to different types of cadavers, thus different radius bones, being used.

### Dorsal tubercle analysis (Figure 5)

The size of the distal dorsal (Lister’s) tubercle and the extent of the EPL groove were evaluated on 100 cadaver specimens and 30 forearms by CT scanning in a study by Clement et al. [8] and Pichler et al. [9]. There were notable variations between the two sets of specimens. Although the tubercle’s height was similar between the cadaver and CT scan studies (3.6 versus 3.3 mm), its length was greater in the cadaver study (18.3 mm) than in the CT scan study (13.2 mm). On the ulnar side, the height between the bottom of the groove and the tip of the tubercle was twice the height (7 mm) in the cadaver study than in the CT scan study (3.4 mm). This variability makes it is very difficult to predict the depth of the EPL groove and height of Lister’s tubercle when performing volar plating for distal radius fractures.

**Figure 5.**
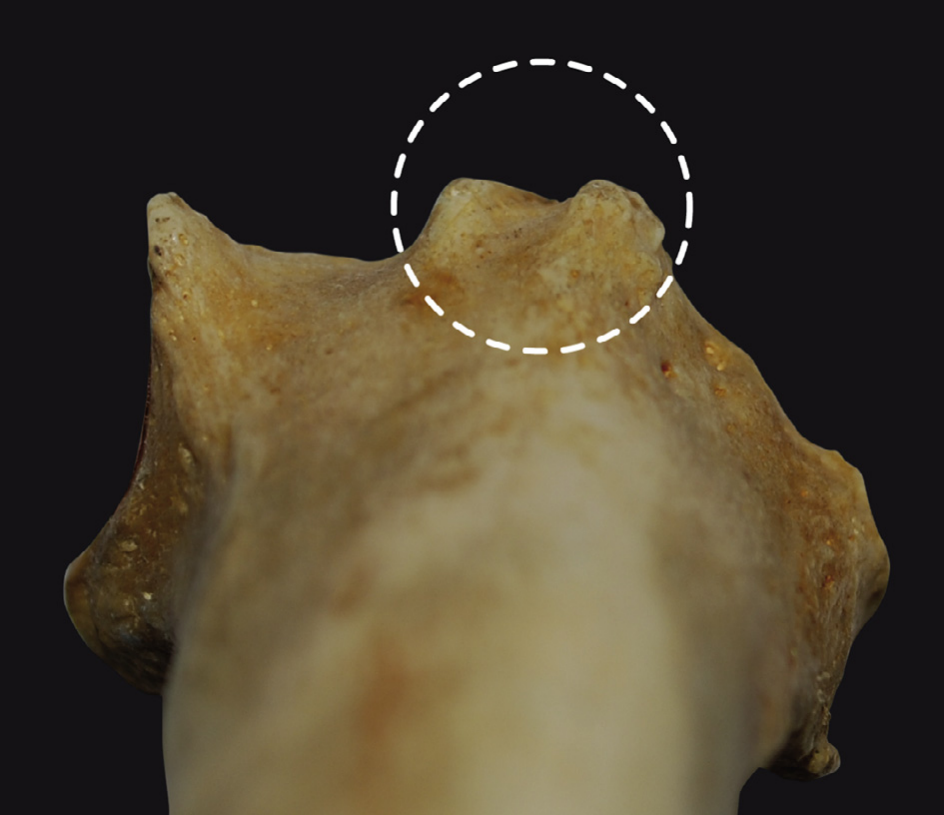
A superior view of the wrist showing the distal dorsal (Lister’s) tubercle and the EPL groove (dotted line).

Gasse et al. [6] also found that the pronator quadratus line and the tip of the dorsal tubercle were separated by about 22 mm. This number is important clinically: the surgeon must be very careful when inserting a screw longer than 22 mm into the distal holes of a plate over the metaphysis, as this often corresponds to the location of the dorsal tubercle.

### Volar cortical angle and wrist columns (Figure 6)

Pichler et al. found variability in the medial radial column in a study with 70 cadaver specimens [10]. Gasse et al. found that the medial radial column was oriented slightly more vertically than the lateral radial column [6]. The volar cortical angle (VCA) was 35° medially and 25° laterally [6]. In a recent CT study by Evans with adult patients (average age of 37 years), a difference between the two columns was noted [11]. The medial slope (34°) was greater than the lateral slope (32°), but this difference was less than in the Gasse study. A gender effect was also present. Women had a greater VCA than men. The average VCA of 32.9 ± 5.1 was greater than the theoretical values typically used.

**Figure 6.**
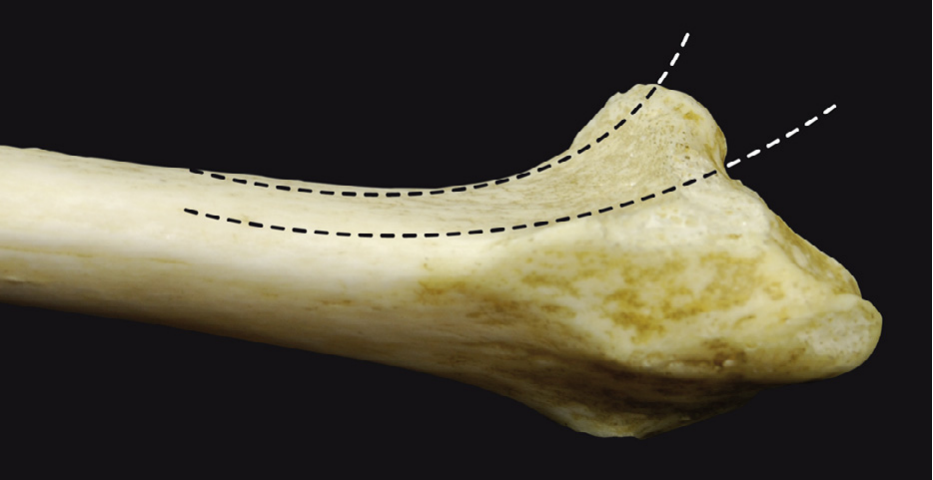
An oblique view of the distal radius showing the wrist columns (medial and lateral).

Buzzell et al. evaluated eight distal radius volar plates and found that the area between the plate and distal radius is very thin and varies by 3–6% [12]. Plates that are currently available on the market have a slope of approximately 155°. However, their slope is constant and does not change over the width of the radius. The radial epiphysis actually has two slopes because of its two columns; this makes it more difficult to develop anatomical plates.

## A fresh look at distal radius biomechanics

The loads experienced by the distal radius vary greatly. Wrist movements during activities of daily living generate loads of nearly 100 N, while finger flexion produces an average of 250 N [13]. Putnam et al. showed that a 10 N grip force translates to an axial force of 26.3 N in the metaphysis of the distal radius. For each 10 N applied, 26–52 N is applied to the distal radius, depending on hand position and radius length [14]. When the grip force reaches 450 N (average for men), a 2410 N load is applied on the radial metaphysis. In certain positions and grips, more than 3000 N can be applied to the distal radius [15]. The load required to break the distal radius is greater than 2500 N [16]. Many advocate grip tightening exercises during rehabilitation should not exceed 169 N, and range-of-motion exercises should not exceed 50% of the implant failure load. However, this is not easy to apply to a patient! Loads that cause fixation system failure range from 55 to 825 N and are directly related to the type of hardware used and its inherent characteristics [15].

Several papers about cortical thickness have been published recently. Mueller et al. analyzed age and gender differences in architectural measurements of bone quality and their correlation to bone mechanical properties in the human radius of an elderly population [17]. Women had considerably thinner trabeculae in subchondral regions. The cortex was relatively thin for both genders in the distal region: 0.41 mm in men and 0.36 mm in women [14]. Dhillon et al. compared the thickness of the volar and dorsal cortices at 0, 5, and 10 mm from the articular surface in 10 cadaver specimens. At each level, the volar cortex was significantly thicker than the dorsal cortex. The mean differences from the articular surface were 0.27 mm, 0.45 mm, and 0.78 mm at 0, 5, and 10 mm, respectively. There were no differences between the thickness of the lateral and medial cortices [18]. This information should be taken into account during volar plating of distal radius fractures [19].

In conclusion, there is no plate or ideal positioning equipment. However, in light of this non-exhaustive review of the literature on the anatomy of the distal radius, there appear to be several recommendations:

- the distal portion of the plate must absolutely not exceed the watershed line and be perfectly at the line of the pronator quadratus,
- the length of the distal epiphyseal screw should not exceed 22 mm (although check the gauge and under fluoroscopy when measures are higher),
- the plates that mimic the best anatomy of the distal radius (latest generation plates) are those that respect the double distal radial and ulnar different curvature,
- the materials used must continue to take into account the important biomechanical forces into play in this anatomical region without losing sight of the need of minimal thickness: balance thinness of the plate/resistance of the material is important.

## Disclosure and conflict of interest

L. Obert is a consultant for FX Solution, Zimmer, SBI, Synthes & Depuy, Medartis, Evolutis, Biotech & wright, Argo. Every other author certifies that he has no commercial associations that might pose a conflict of interest in connection with the submitted article.

The authors certify that they have no conflict of interest in connection with this article.

## References

[1] Obert L et al. (2012) Anatomy and biomechanics of distal radius fractures: a literature review. Chir Main 31(6), 287–297. 23177906 10.1016/j.main.2012.08.010

[2] Herzberg G et al. (1998) Anatomie du radius distal. Cahiers d’enseignement de la SOFCOT. Paris, Expansion scientifique Publications, 14–27.

[3] Windisch G et al. (2001) Capsular attachment to the distal radius for extracapsular placement of pins. Surg Radiol Anat 23(5), 313–316. 11824129 10.1007/s00276-001-0313-6

[4] Windisch G et al. (2007) Promontory of radius: a new anatomical description on the distal radius. Surg Radiol Anat 29(8), 629–633. 17928939 10.1007/s00276-007-0264-7

[5] Nelson D (2013) Anatomy notes and their clinical significance for the volar approach By David L. Nelson, MD, http://www.davidlnelson.md/articles/Radius_Anatomy_Annotated.htm.

[6] Gasse N et al. (2011) Anatomical and radiological study applied to distal radius surgery. Surg Radiol Anat 33(6), 485–490. 21136059 10.1007/s00276-010-0754-x

[7] Imatani J et al. (2012) An anatomical study of the watershed line on the volar, distal aspect of the radius: implications for plate placement and avoidance of tendon ruptures. J Hand Surg 37(8), 1550–1554. 10.1016/j.jhsa.2012.05.011 22835584

[8] Clement H et al. (2008) Morphometric analysis of lister’s tubercle and its consequences on volar plate fixation of distal radius fractures. J Hand Surg 33(10), 1716–1719. 10.1016/j.jhsa.2008.08.012 19084168

[9] Pichler W et al. (2009) Computer tomography aided 3D analysis of the distal dorsal radius surface and the effects on volar plate osteosynthesis. J Hand Surg Eur 34(5), 598–602. 10.1177/1753193409101471 19959446

[10] Pichler W et al. (2008) Various circular arc radii of the distal volar radius and the implications on volar plate osteosynthesis. Orthopedics 31, 12. 10.3928/01477447-20081201-18 19226073

[11] Evans S et al. (2014) Distal volar radial plates: how anatomical are they? Orthop Traumatol Surg Res 100(3), 293–295. 24662604 10.1016/j.otsr.2013.11.014

[12] Buzzell JE et al. (2008) Precontoured fixed-angle volar distal radius plates: a comparison of anatomic fit. J Hand Surg 33(7), 1144–1152. 10.1016/j.jhsa.2008.02.029 18762111

[13] Osada D et al. (2003) Comparison of different distal radius dorsal and volar fracture fixation plates: a biomechanical study. J Hand Surg 28(1), 94–104. 10.1053/jhsu.2003.50016 12563644

[14] Putnam MD et al. (2000) Distal radial metaphyseal forces in an extrinsic grip model: implications for postfracture rehabilitation. J Hand Surg 25(3), 469–475. 10.1053/jhsu.2000.6915 10811751

[15] Mathiowetz V et al. (1985) Grip and pinch strength: normative data for adults. Arch Phys Med Rehabil 66(2), 69–74. 3970660

[16] Augat P et al. (1998) Distal radius fractures: mechanisms of injury and strength prediction by bone mineral assessment. J Orthop 16(5), 629–635. 10.1002/jor.1100160517 9820289

[17] Mueller TL et al. (2009) Regional, age and gender differences in architectural measures of bone quality and their correlation to bone mechanical competence in the human radius of an elderly population. Bone 45(5), 882–891. 19615477 10.1016/j.bone.2009.06.031

[18] Dhillon SS et al. (2007) Anatomical study comparing the thickness of the volar and dorsal cortex of cadaveric adult distal radii using digital photography. Arch Orthop Trauma Surg 127(10), 975–977. 17619198 10.1007/s00402-007-0394-8

[19] Obert L et al. (2013) Fixation of distal radius fractures in adults: a review. Orthop Traumatol Surg Res 99(2), 216–234. 23518070 10.1016/j.otsr.2012.03.023

